# Age-Friendly Online Training Programs for Long-Term Services and Supports Staff to Improve Care for Older Adults

**DOI:** 10.1093/geroni/igaa057.1719

**Published:** 2020-12-16

**Authors:** Linda Edelman, Kara Dassel

## Abstract

The purpose of our Geriatric Workforce Enhancement Program is to provide geriatric and primary care education and training to long-term care (LTC) providers and staff, health professions students and community members. Our LTC partners and the communities we serve are often very rural and travel to urban areas for training can be difficult. Therefore, we have developed four online training that are offered free to our partners and rural communities statewide. These programs are designed to integrate the aims of the Age-Friendly 4M’s model (i.e., What Matters, Mobility, Medication, Mentation). The LTC nurse residency program provides gerontological nursing and inter-professional leadership training (all 4M’s), in a synchronous online environment. The asynchronous Alzheimer’s Disease and Related Dementias training modules educate LTC staff and family caregivers about types, diagnosis and care of older adults with dementia (Mentation and Medication). The asynchronous Opioid Use in LTC modules were developed with partners to deliver live at LTC staff trainings about opioid stewardship (Medication). The LTC Learning Communities are monthly tele-health sessions for inter-professional LTC teams to discuss current issues and propose solutions (all 4M’s). We have successfully leveraged different synchronous and asynchronous online modalities to increase educational opportunities for formal and informal caregivers, including those in rural areas whose educational opportunities are geographically limited. To date our programs have reached over 500 individuals across our state, increasing knowledge about geriatric concepts, communication and team leadership. Moving forward, we will continue to develop and refine educational programs that promote the Age-Friendly geriatric-focused health care.

